# Framework to Assist Stakeholders in Technology Evaluation for Recovery (FASTER) to Mental Health and Wellness

**DOI:** 10.1186/s12913-025-12418-0

**Published:** 2025-04-30

**Authors:** Smisha Agarwal, Madhu Jalan, Rachel Hill, Emily Pantalone, Johannes Thrul, Ritu Sharma, Holly C. Wilcox, Karen A. Robinson

**Affiliations:** 1 Center for Global Digital Health Innovation, Johns Hopkins Bloomberg School of Public Health, Baltimore, MD USA; 2 Department of International Health, Johns Hopkins Bloomberg School of Public Health, 615 N Wolfe Street, Baltimore, MD 21205 USA; 3 Department of Mental Health, Johns Hopkins Bloomberg School of Public Health, Baltimore, USA; 4 Sidney Kimmel Comprehensive Cancer Center at Johns Hopkins, Baltimore, MD USA; 5 Centre for Alcohol Policy Research, La Trobe University, Melbourne, Australia; 6 Johns Hopkins University Evidence-Based Practice Center, Johns Hopkins Medicine, Baltimore, MD USA; 7 Department of Medicine, Division of General Internal Medicine, Johns Hopkins University School of Medicine, Baltimore, MD USA; 8 Department of Health Policy and Management, Johns Hopkins Bloomberg School of Public Health, Baltimore, MD USA; 9 Department of Epidemiology, Johns Hopkins Bloomberg School of Public Health, Baltimore, MD USA

**Keywords:** Mental Health, Digital Health, Technology Evaluation, Effectiveness, Safety

## Abstract

Mental health mobile applications (apps) have the potential to expand the provision of mental health and wellness services to underserved populations. There is a lack of guidance on how to choose wisely from the thousands of mental health apps without clear evidence of safety, efficacy, and consumer protections. We propose the Framework to Assist Stakeholders in Technology Evaluation for Recovery (FASTER) to Mental Health and Wellness to support agencies and individuals working in mental health, as well as users of mental health apps, in making informed decisions recommending the use of, or using mental health and wellness apps. The framework was developed through a systematic process including a review of existing frameworks and the literature, interviews with key informants, public stakeholder feedback, and iterative application and refinement of the framework to 45 apps. It comprises three sections: Section 1. Risks and Mitigation Strategies, assesses the integrity and risk profile of the app; Section 2. Function, focuses on descriptive aspects related to accessibility, costs, developer credibility, evidence and clinical foundation, privacy/security, usability, functions for remote monitoring of the user, access to crisis services, and artificial intelligence (AI); and Section 3. Mental Health App Features focuses on specific mental health app features, such as journaling and mood tracking. The framework facilitates an assessment of the level of risk posed by the app against the evidence on the effectiveness of the app and its safety features, recognizing that given vast variations in health apps, a ‘one size fits all’ approach is likely to be insufficient. Future application, testing, and refinements may be required to determine the framework’s suitability and reliability across multiple mental health conditions.

**Supplementary Information:**

The online version contains supplementary material available at 10.1186/s12913-025-12418-0.

## Background

Among adults aged 18 or older in the United States, the prevalence of ‘mental illness in the past year’ increased from 17.7 percent (or 39.8 million people) in 2008 to 23 percent (or 58.7 million people) in 2023 [1]. Of this latter population, 27.1 million did not receive mental health treatment and of these 23.8% had an unmet need for mental health services in the past year. The demand for mental health services far outweighs existing resources and the capacity of the healthcare system to meet these needs [2]. Several factors drive limited access to mental healthcare services, including affordability, availability of mental health care providers, acceptance of insurance by mental healthcare providers [3], and the stigma associated with mental health care [4]. Moreover, there are racial and socio-demographic disparities in access to mental health care- Black and Latino Americans are half as likely to access mental health care compared to non-Latino whites [5]. Isolation and anxiety related to the COVID-19 pandemic has amplified the challenges around mental health care access. In December 2021, the U.S Surgeon General Issued an advisory outlining the unprecedented impacts of the pandemic on the populations’ mental health, especially among children, adolescents, and young adults. The advisory also outlined the disproportionate impacts on racial and ethnic minorities, LGBTQ + youth, low-income youth, immigrant households, those with disabilities, youth involved in the child welfare and juvenile systems, and homeless populations [6].

Considering numerous barriers to mental health care including the shortage of mental health care providers in many settings, digital mental health interventions, accessible through the internet on tablets and mobile phones have the potential to provide much needed access to mental health services. Mental health apps can be used by individuals on their own or may complement treatment plans from health care providers. Mental health apps may especially serve to overcome barriers to access for marginalized and underserved populations [7]. Such technology-driven mental health interventions may offer a scalable and accessible augmentation or bridge to traditional care. Research on efficacy and effectiveness of mental health apps has been emerging across mental health conditions and contexts, including self-management of acute symptoms and well-being, as well as early evidence on clinical management of chronic psychiatric conditions [8]. Recent meta-analyses of apps targeting anxiety and depression suggested moderate efficacy of apps in reducing the symptoms of anxiety and depression [9–11]. Yet despite emerging evidence, few patients receive a recommendation to use mental health apps by mental health professionals or other healthcare providers [12].

One factor that has contributed to the unrealized potential of mental health apps is their rapid and unregulated proliferation that has resulted in a lack of confidence in the safety and efficacy of these apps, and subsequently their lack of uptake and sustained use among patients. The US Food and Drug Administration (FDA) announced a “precertification” program for mobile apps, to supply information about the app development organizations’ quality control process for software [13]. However, this program does not support an evaluation of whether the app improves healthcare outcomes, leaving a critical gap in the safe and effective use of such apps in clinical practice. Without federal agencies requiring review and approval of mental health apps or a validated assessment of mental health apps, clinicians may feel ill-equipped to recommend an app to a patient.

Patients, on the other hand, may decide to use a mental health app largely based on ratings and consumer reviews, with inadequate understanding of whether such apps are evidence-based. Research suggests that consumer ratings are a poor indication of an app’s effectiveness, and found that several apps did not have an appropriate response when a user provided potentially concerning health information that would warrant escalation in a traditional healthcare environment [14]. Apps that claim to provide a diagnosis or that target individuals who may be vulnerable due to their mental health condition or age, can pose a serious risk outside of the bounds of clinical care [15]. Mental health apps should offer direct linkage to working crisis lines. Concerningly, there have been more than 2 million downloads of mental health apps that either entirely lack or contain inaccurate suicide crisis phone numbers [16]. Moreover, users may consent to the use of the app without understanding privacy agreements or data sharing schemes and may unknowingly share financial or private health information [17]. An assessment of health and wellness apps suggested that 95 percent posed some threat to privacy infringement of the users [18].

The demand for and scalability of mental health apps coupled with growing concerns over safety, privacy, and the effectiveness of mental health apps have created the need for an assessment framework that can be systematically applied to inform selection of mental health apps by organizations, clinicians and patients [13]. There are existing frameworks used to evaluate digital health apps, including some that focus on mental health apps [19–23].

Examples of frameworks for the assessment of general health app assessment include Healthy Living Apps Guide, Digital Technology Assessment Framework from the National Health Service UK, and Digital Therapeutics Alliance. Examples of frameworks that are focused on mental health app assessment include One Mind Psyber Guide [19], APA App Advisor (American Psychiatric Association) [20], Kaiser Permanente [21], VeryWellMind [22], and Health Navigator [23]. Other notable frameworks reviewed include M-Health Index and Navigation Database (MIND) and the end-user version of the Mobile Application Rating Scale (uMARS). MIND is an operational and flexible framework based on the American Psychiatric Association App Assessment Framework, which includes 105 questions that have been harmonized from 79 frameworks. The end-user version of the Mobile Application Rating Scale (uMARS) provides a comprehensive set of questions about app engagement and usability [33].

However, most existing frameworks are geared towards evaluating specific aspects of health apps (e.g., such as usability), and are not tailored towards an assessment of their potential risks and evidence on clinical benefits. To address this gap, the Agency for Healthcare Research and Quality (AHRQ) supported the development of the Framework to Assist Stakeholders in Technology Evaluation for Recovery (FASTER) to Mental Health and Wellness to guide mental health and wellness app selection based on safety, effectiveness and features important to patients and providers. The goal of FASTER to Mental Health and Wellness is to support agencies and individuals working in mental health, as well as users of mental health apps, in selecting mental health and wellness apps. We expect that this framework will be applied by intermediary mental health advocate groups and agencies that have the capacity to train personnel to use this framework to evaluate mental health apps and employers or insurance companies that might have an interest in reimbursing for the use of certain health apps. The results and summary conclusions of such app assessments using FASTER will be valuable to healthcare professionals before they recommend or prescribe apps to patients, and to patients/users/caregivers in search of mental health and wellness apps. The framework might also inform and guide app developers in the development of apps. Here, we describe the development process, framework components, and its intended use.

## Methods

The FASTER to Mental Health and Wellness framework was developed following a four-step process outlined in a protocol available on AHRQ’s Effective Health Care website [24], and provided in more detail in the full report, and in Supplement Figure A. Here we briefly summarize the process.

1. Review and abstraction of existing frameworks: Existing health app frameworks were identified through a systematic search of PubMed, as well as guided by the authors knowledge of health apps-related frameworks. Peer-reviewed literature that described frameworks to evaluate health apps, specific aspects of health apps (e.g., usability), and mental health apps were included. Documents from federal drug agencies from the US (FDA), UK (National Institute for Health and Care Excellence [NICE]), and Germany (Federal Institute for Drugs and Medical Devices) provided additional information on federal regulation of apps. Relevant items were abstracted from 11 frameworks and consolidated. Items that were clear to understand and could be applied by individuals without specialized mental health or technology training were retained. Additionally, items that could be applied without significant time and research resources were retained. For example, searching bibliographic research databases such as PubMed or MEDLINE for evidence was considered beyond the scope of how this framework is intended to be used. Therefore, items that would require such a search were omitted. This yielded a total of 300 questions.
2. Identification of critical needs to assess mental health and wellness apps: Four rounds of key informant interviews (KI) with a total of 12 stakeholders were conducted. The first two set of interviewees included clinicians with a background in mental health, primary health care, and emergency medicine, and payors. A third round of interviews was conducted with app developers and mental health providers with app development expertise. The purpose of these interviews was to identify the essential components to include in a mental health app framework to best guide selection of a mental health app. The fourth round of interviews included family members of individuals living with mental illness.
3. Development of a draft framework: As a next step, we developed additional items to address the omissions identified in the existing frameworks based on the analyses and KI feedback. New thematic areas included risk assessment of apps, cultural appropriateness, use of machine learning/artificial intelligence (AI), informed consent, and inclusion of mental health app features and crisis resources to support mental health and wellness. Existing items, abstracted from other frameworks, were modified, where necessary, for clarity and ease of application. A training guide was simultaneously developed to facilitate broad uptake of the framework. For the assessment of risks posed by the apps, guidance was utilized provided by agencies such as National Institute for Health and Care Excellence (NICE) [25] and various FDA documents including Clinical Decision Support and Software as a Medical Device guidance [26–28]. For the development of criteria on the use of AI, literature on issues of safety in the use of AI for health apps was reviewed and an ethicist specializing in machine learning was consulted.
4. Refinement of the framework: The draft framework was tested and iteratively refined through one pre-pilot and six pilot rounds where the framework was applied to mental health and wellness apps across a range of 15 mental health conditions defined by the Diagnostic and Statistical Manual of Mental Disorders (Supplementary Table D). We used mental health symptoms and diagnostic categories from the DSM-5 to guide our search and selection of mental health apps. We cross-checked the main diagnostic categories with mental health conditions addressed by current mental health apps and included those addressed by at least one app. In addition to the DSM-5 categories, we added categories for Self harm; Mental wellness; and Other mental disorders. A convenience sample of 10 apps were chosen as a pilot. After that 35 apps were chosen in a serialized fashion from the randomized list of apps categories. A team of 11 reviewers were trained over 2 h. Reviewers included undergraduate public health major students, and graduate public health students with an interest in mental health and health care technology. In total 45 apps were reviewed between May-December 2021, each by at least 2 reviewers. In each round, items that did not have perfect agreement were discussed, and items were either modified or training guidance notes were provided so that each item could be applied in a standardized way.

The development of the framework was not considered as human subjects’ research.

## Results

**Fig. 1:**
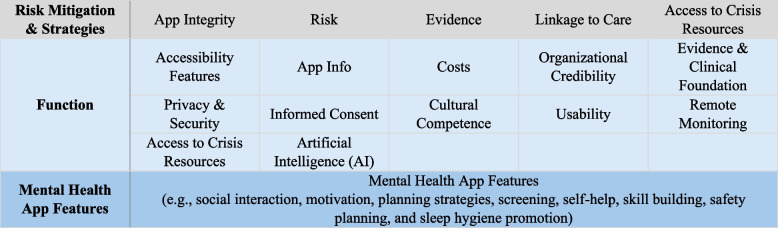
FASTER to Mental Health and Wellness: sections and categories of items

The Framework to Assist Stakeholders in Technology Evaluation for Recovery (FASTER) to Mental Health and Wellness comprises three sections (Fig. 1) with an initial and concluding set of administrative questions: “Section 1: Risks and Mitigation Strategies”, “Section 2: Function” and “Section 3: Mental Health App Features”. Within each of these sections, there are a series of items related to the assessment of specific categories considered critical based on the literature review and key informant interviews. Details about the specific items are in Supplementary materials C.

### Intended use

Evaluators who use this framework should be individuals with some background in technology and mental health, but do not need to be experts in technology or mental health. The results from applying the framework will be valuable to healthcare professionals as they recommend apps to patients, and to patients/users/caregivers who are in search of a mental health or wellness app. The framework might also inform and guide app developers in the development of apps.

The framework is intended to be applied to assess apps whose primary function is to support mental health and wellness through content and resources within the app. It is not appropriate to use this framework to evaluate apps whose primary function is to facilitate telemedicine (e.g., link users/patients to a mental health professional), or health apps that might contain supplemental content to support wellness (e.g., a weight loss app that has resources for mindfulness). The approach for summarizing the risk levels based on Sect. 1 of the framework is available is Supplementary section B.

### Introduction to the Framework to Assist Stakeholders in Technology Evaluation for Recovery (FASTER) to Mental Health and Wellness

#### Section 1: Risks and mitigation strategies

This section facilitates an assessment of the app risk profile and serves to flag apps that do not meet basic safety, evidence, and security checks. Supplementary section C, Sect. 1 details the specific questions within each item.

- **App Integrity**: Questions under this category aim to assess whether an app uses personal health and financial information appropriately, and that the app has a legal commitment to user privacy and security. The questions also assess whether an app has been endorsed or is being used by a trusted federal agency (e.g., National Institutes of Health), or non-government body (e.g., American Psychiatric Association) which would reinforce credibility, as these institutions exercise due diligence before endorsing or making an app available to their users. Based on the responses, two integrity levels are assigned:
  *High integrity*: If the app has been updated in the previous 6 months, ensures privacy and security of the user’s data (or/and provides disclaimers and warnings), and/or if the app has been endorsed by a trusted organization.
  *Low integrity*: If the app has not been updated in the previous 6 months and/or provides no privacy and security statement, and/or provides no disclaimers and warnings.
- **Risk**: The level of risk posed by the app is determined based on a set of questions related to the goals of the app (e.g., standalone treatment, support through building coping skills, etc.), the target audience (e.g., minors), and severity of the mental health condition.
- **Evidence**: Questions in the Evidence category help determine whether the app has a clinical research foundation. The greater the risk of an app, the greater the burden of evidence. For apps that pose a higher level of risk, the framework requires that there are robust studies assessing the efficacy and risks posed by the apps.
- **Linkage to Care**: Questions evaluate the linkages to a healthcare provider who can monitor the patient’s app use to enhance care manually or through app data shared to their electronic health record (EHR) system. If the app could pose a higher level of risk, the framework requires that it also provide resources for linkage to care.
- **Access to Crisis Services**: Questions in the Access to Crisis Services category evaluate whether the app provides direct or indirect linkage to crisis services (e.g., dial 988 to reach the National Suicide Prevention Lifeline in the United States).

#### Section 1 summary

Questions in the Risk Assessment category assess the risks posed by a mental health app. The “level of risk” as determined by responses to questions (details of how risk level is assessed is provided in Supplement B) is balanced against the mitigation strategies to counter this risk based on questions related to the available evidence to support the approach, clinical oversight and linkage to care, and access to crisis services. This risk-based approach is guided by the International Medical Device Regulators Forum “Software as a Medical Device (SaMD): Clinical Evaluation [29]. For example, if the app is intended for use by populations with a condition that results in severe cognitive impairment, or by children, or if the app intends to provide standalone treatment, it may pose a higher risk to the target population. To ensure safety of the target populations, this section assesses whether sufficient evidence exists to support the app’s stated goals, and whether the linkages to care/access to crisis services provided within the app are appropriate given the potential risks (Table 1).

**Table 1:** Risk levels and mitigation strategies

Risk level	Mitigation strategies
Risk Level 1: Minimal Risk	No requirement for providing evidence or for linkage to care. For example, apps aimed at supporting mindfulness practices would fall into this category
Risk Level 2: Some Risk	Requires some research support regardless of the experimental design. The app should also leverage an evidence-informed theory to guide its approach. Additionally, it should facilitate remote sharing of information with a provider and provide the user with information on a crisis hotline or other resources. For minors, developmentally disabled adults, and older adults who have become incapacitated, the app should require legal guardian or caregiver permission and facilitate sharing of information with them
Risk Level 3 Considerable Risk	Requires research support with at least one or more **randomized controlled trial** that show evidence of impact. The app should also leverage an evidence-informed theory to guide its approach. Additionally, it should facilitate remote sharing of information with a provider and provide the user with information to access a crisis hotline or other resources. For minors, developmentally disabled adults, and older adults who have become incapacitated, the app should facilitate sharing of information with a legal guardian or caregiver

#### Section 2: Function

This section is focused on descriptive aspects of an app. These criteria are intended to (1) describe features offered by the app for users to assess its fit for their therapeutic and wellness needs (2), systematically catalogue the functions of the app, so that users may choose an app based on the functionality (3), present users with additional details about the potential risks related to the use of the app so that they can be informed consumers. Specific questions are outlined in Supplementary material C, section 2.

- **Accessibility Features**: The questions assess whether an app has features that facilitate easier use of the app by individuals with disabilities. Accessibility features assessed include text adjustment, colorblind color scheme features, text-to-speech, availability of transcriptions/captions, configurability of keyboard shortcuts and availability of a screen reader.
- **App Information**: This section captures details about the platform required by the app (e.g., iOS, Android), and users’ reviews and ratings.
- **Costs**: Increasingly, apps have complex pricing models which, especially in the case of a vulnerable user base with mental health impairments, may pose risks. The questions assess whether costs associated with the app are provided upfront, and whether the pricing model (e.g., free, onetime cost, in-app purchases, subscription model, reimbursable by healthcare insurance, etc.) is clearly presented.
- **Organizational Credibility**: The questions assess the reputation of the organization that has developed the app based on the type of organization (governmental, for-profit, not-for profit, etc.) and whether there are any documented consumer complaints against the app developing organization.
- **Evidence & Clinical Foundation**: The question related to evidence in this category goes beyond what was assessed in Sect. 1 to assess whether the app addresses its stated goals.
- **Privacy/Security**: The questions to assess privacy and security focus on whether any claims of Health Insurance Portability and Accountability Act (HIPAA) or other analogous national standards for protected health information (PHI) have been made, whether the app is transparent about how user data are used, and whether the app uses industry standards to share data with EHRs.
- **Informed Consent**: Informed consent is a process for getting permission before conducting some form of research using health data, or prior to sharing the users’ health and related information. Most apps have a disclosure list that is long and hard to understand. There are best practices for ensuring that information is presented in a way that is understandable by users [30–32]. The questions in this category evaluate whether the app follows these best practices.
- **Cultural Competence**: Cultural competence is defined as the ability to understand, appreciate, and account for different cultures or belief systems based on race, ethnicity, sexual orientation, income strata, religious beliefs, etc. The questions in this category assess whether the app is targeted at, or inclusive of, specific population groups and cultures. If the app is targeted at a specific cultural group, the criteria assess whether the app has been tested in that group. The criteria also assess the use of gender inclusive language, and evidence of effectiveness in a non-white population.
- **Usability**: Usability can be described as the capacity of an app to provide conditions for its users to perform safely, effectively, and efficiently. The usability criteria for this framework were adapted from the UMARS framework [33]. The usability assessment has some objective criteria (e.g., offline use, languages supported by the app, etc.), as well as criteria that might introduce some level of subjectivity from the evaluator (e.g., design of the app layout, clarity of the content).
- **Functions for Remote Monitoring of the User**: Remote patient monitoring is a technology to enable monitoring of patients outside of conventional clinical settings, such as in the home [34]. For mental health apps, the provider may receive an alert about their patient's health, or they may be able to access the patient’s health indicators from within the app. To enable remote monitoring, apps need to adhere to established data standards for interoperability to safely exchange health data, including with wearable devices that may be used to monitor vital parameters or behaviors. Questions assess how data are shared for remote monitoring, the availability of two-way communication with providers, and data sharing capabilities with wearable devices.
- **Access to Crisis Services**: An additional question related to access to crises services assess whether the app has additional functionality to automatically link the user to a provider or to a crisis line in case of an emergency.
- **Artificial Intelligence**: Increasingly, mental health apps are incorporating or claiming to incorporate artificial intelligence (AI) for the purposes of customizing feedback and interventions and identifying mental health risks [35]. This area is rapidly emerging, and the questions relate to whether the app claims to use AI, and how.

#### Section 3: Mental health app features

This is a specialized section of the framework focused on the availability of therapeutic features and skill-building approaches that are typically employed by mental health care providers to support their patients (Supplementary Material C, Sect. 3). The assessment of apps for these functionalities will facilitate cataloguing of functions from which users may benefit and find engaging. Questions pertain to the availability of two-way communication with therapists and coaches through text message, audio or video features, availability of group therapy services, live support by a coach, and concierge mental health services. Additionally, the questions also seek to address the comprehensiveness of functionality related to mindfulness, journaling, psychoeducation, building coping skills, self-screening, safety planning, sleep regulation, chatbot support, family/caregiver support, peer group interaction, etc.

## Discussion

FASTER to Mental Health and Wellness is aimed at facilitating the selection of apps for mental health support through standardized evaluation, screening, and classification of apps. Several of the criteria have been extracted or modified from existing frameworks in the app evaluation and mental health space, and new criteria have been developed and tested to address emerging concerns in the use of apps for mental healthcare. This framework provides two novel areas of contribution. First, Sect. 1 of the framework facilitates an assessment of the level of risk posed by the app against the evidence on the effectiveness of the app and its safety features, recognizing that given vast variations in mental health apps, a ‘one size fits all’ approach is unlikely to be sufficient. This framework provides a level of assessment that is tailored to the stated goals of the app with the goal of empowering end users with critical information to support appropriate selection of an app. Second, this framework facilitates systematic cataloguing of a wide range of functionalities such as sleep journaling, and skill building that are increasingly being embedded in apps to support patients. Such a catalogue of app functions can assist patients and providers in the selection of apps that best fit their individualized needs.

Assessment and standardization of mental health apps poses some unique challenges that we anticipate will continue to require attention. Many mental health symptoms are transdiagnostic, and typically apps may aim to support alleviation of a symptom rather than the disorder. Several mental health apps may aim to target symptoms such as anxiety or insomnia which are common across several mental health conditions. As it stands, the framework assesses the risks posed by an app based on the health condition it targets and the level of functional impairment an average patient might experience due to their health condition. However, an individual’s mental health condition can deteriorate rapidly, which also changes the potential risks from the use of a mental health app. The framework proposes mitigation of this risk through linkage to a healthcare provider and other caregivers. Further refinement of the framework may be needed to address applicability across apps that target transdiagnostic symptoms. Additional criteria may be needed to account for potential harm or iatrogenic impacts of an app, based on the severity or other characteristics of specific mental health conditions or the culture and characteristics of the user.

We expect that this framework may also benefit from updates to reflect emerging areas in the use of health apps. As new governance and regulations for software as a medical device are formulated, the framework should be adapted to include those. Additionally, developments in our understanding of prerequisites for apps from a privacy/security perspective, as well as rapid innovation in the digital health and AI space will need to be incorporated as additional criteria. In future versions of the framework, it will be important to add greater input from commercial app developers as they can provide insight regarding the app roadmap and challenges in commercializing health apps. It would also be critical to test this framework by applying it to apps classified as digital therapeutics that require prescriptions.

Ultimately, to facilitate the adoption and sustainability of this framework, it would be necessary to have a centralized system in place to update the framework as mental health app technology advances and to train personnel to apply this framework to screen apps. The results of the review of apps using this framework would ideally be hosted as an interactive webpage that can be used by patients and mental health advocacy agencies. To further facilitate appropriate use of mental health apps in clinical and public health contexts, a systematic way to provide education is necessary across the healthcare ecosystem to convey to end-users and licensed mental health professionals and other clinicians, the potential benefits and risks of such health apps as technology continues to advance. This framework provides foundational guidance towards that goal.

## Supplementary Information

Supplementary file 1.

## Data Availability

The datasets used and/or analysed during the current study are available from the corresponding author on reasonable request.
