# Supplementary material for: Framework to Assist Stakeholders in Technology Evaluation for Recovery (FASTER) to Mental Health and Wellness
Source: BMC Health Serv Res. 2025 Apr 30;25:623. doi: 10.1186/s12913-025-12418-0 (PMC12044955; doi:10.1186/s12913-025-12418-0)
Supplement: Supplementary file 1 [file 12913_2025_12418_MOESM1_ESM.docx]

**Supplementary Material**

**Supplement Figure A : Process for the development of the FASTER Framework**

**Supplementary Table B. Risk levels and mitigation strategies**

| **Risk level** | **Mitigation strategies** |
| --- | --- |
| Risk Level 1: Minimal Risk | No requirement for providing evidence or for linkage to care. For example, apps aimed at supporting mindfulness practices would fall into this category. |
| Risk Level 2:  Some Risk | Requires some research support regardless of the experimental design. The app should also leverage an evidence-informed theory to guide its approach. Additionally, it should facilitate remote sharing of information with a provider and provide the user with information on a crisis hotline or other resources. For minors, developmentally disabled adults, and older adults who have become incapacitated, the app should require legal guardian or caregiver permission and facilitate sharing of information with them. |
| Risk Level 3  Considerable Risk | Requires research support with at least one or more **randomized controlled trials** that show evidence of impact. The app should also leverage an evidence-informed theory to guide its approach. Additionally, it should facilitate remote sharing of information with a provider and provide the user with information to access a crisis hotline or other resources. For minors, developmentally disabled adults, and older adults who have become incapacitated, the app should facilitate sharing of information with a legal guardian or caregiver. |

**Supplementary material C - Framework**

**Administrative Questionnaire**

This section contains introductory questions about the app to be reviewed. The questions are mostly factual, rather than evaluative. Some of these questions may make it into the final Assessment Framework, based on the information we gather from the pilot.

**Q 1. Reviewer Name**

________________________________________________________________

**Q 2. Date of Evaluation**

_______________________________________________________________

**Q 3. App Name**

________________________________________________________________

**Q 4. App/ Company Website**

*This should only be a specific website developed by the app developer and not their pages on social media sites, such as Instagram or Facebook.*

________________________________________________________________

**Q 5. Country of Origin**

*This information can be found in the Terms & Conditions on the website or in the app.*

________________________________________________________________

**Q 6. App Version Number**

*This information can be found on the app and from the App Store.*

________________________________________________________________

**Q 7. Platform used for assessment by the reviewer**

_______________________________________________________________

**Q 8. OS Version Number on Evaluating Device**

*For Android go to: Settings > About Phone > Software Information;*

*For iOS go to: Settings > General > Software Version.*

________________________________________________________________

**Q 9. Describe the goal of the app in your own words. Do not use any of the marketing phrases/lingo used by the app developer.**

*This should be based on the app description on the website.*

________________________________________________________________

**Q 10. Has the app been approved by any regulating authority, such as FDA?**

*This information can be found on the app developer website.*

________________________________________________________________

**Q 11. Does the app have multiple revenue models, such as freemium, in-app purchases, etc.?**

- - Yes
  - No

**Q 12. If the app has multiple versions (e.g., free, freemium, paid), which version did you evaluate? If the app had only one revenue model, please choose “not applicable” here.**

- - Free
  - Paid version
  - Paid by insurance agency, employer, or healthcare provider
  - Not applicable
  - Unable to assess

**Q 13. Does the app require prior authorization from a healthcare institution/insurance provider/college/employer or other institution for access?**

- - Yes
  - No
  - Unable to assess

**Q 14. If you were unable to assess the app, please specify the reason.**

Note: Questions about privacy, security, and informed consent are covered in both Section 1 and Section 2 of the framework. Please make a note of all the disclaimers, warnings, and privacy and security statements and agreements when you first log into the app, so you are able to answer the questions that come later in the framework.

**Section 1: Risks and Mitigation Strategies**

Section 1 aims to evaluate the potential risk posed by the app, and evidence of efficacy, and safety features. The burden of evidence to prove efficacy and safety is dependent on the goals of the app, the target audience, and the severity of the mental health condition.

*The categories of questions in this section are App Integrity, Risk Assessment, Evidence, Linkage to Care, and Access to Crisis Services. If apps do not meet the threshold for safety and credibility, that is flagged in the assessment.*

**A. App Integrity**

The information requested in these questions can be found on the App Store, the app’s website, or via Google Search.

**1. Was the version of the app you are reviewing updated in the last 6 months?**

- - Yes
  - No
  - Unable to assess

**2. Does the app provide a privacy and security agreement to be reviewed and agreed to by the user?**

*The agreement should be accessible through a link on the app or on the mobile app. Reviewers should not look for this information in other locations, such as the developer’s website.*

- - Yes, there is a privacy and security agreement to be agreed to by the user
  - No, there is not a privacy and security agreement
  - Unable to assess or unable to access

**3. Does the app provide warnings and disclaimers (e.g., the limitations of the app related to medical liability)?**

*Reviewers should look for this information on the mobile app ONLY.*

- - Yes, warnings and/or disclaimers are provided
  - No, warnings and/or disclaimers are not provided
  - Unable to assess
  - Not applicable

**4. Has the app been endorsed by, or is it being used by, a government agency or trusted mental health professional association?**

*Endorsement and/or usage helps us establish the credibility of the app.*

*Endorsement means to give one's approval to, especially officially by an organization. An app merely being reviewed by the American Psychological Association (APA), or a similar organization, does not mean it has been approved by them. Endorsement should be for the app and not the company developing the app or the domain (e.g., teletherapy). Self-reported endorsements are acceptable.*

*Usage by an institution signifies that an app is made available to all its members. For example, Veterans Affairs uses iBlueButton to deliver critical health care information to veterans.*

*Examples of government agencies include federal, state and local agencies such as CMS, Veterans Affairs, Virginia Department of Health. Examples of trusted mental health professional associations include Substance Abuse and Mental Health Services Administration (SAMHSA), American Psychological Association (APA), and National Alliance on Mental Illness (NAMI).*

- - Yes, the app has been endorsed by one or more mental health associations, government agencies, or non-government bodies (examples may include but are not limited to government agencies, such as Centers for Medicare & Medicaid Services (CMS) or Veterans Affairs (VA); non-government bodies, such as APA; and insurance or healthcare institutions)
  - No, the app has not been endorsed
  - Unable to assess, please specify reason: __________________________________________

**App Integrity Assessment**

| **Question** | **Response** | **App Integrity Level** |
| --- | --- | --- |
| **Does the app satisfy the Integrity Assessment?**  **Please pick only 1 response.** | - Responses to questions 1-3 are all **Yes** OR response to question 4 is **Yes** | App Integrity is “High” |
|  | - Response to any of the questions 1-3 is **No** AND response to 4 is **No** | App Integrity is “Low” |

**B. Risk**

This category aims to determine the level of risk posed by the app. For example, if the app is used by someone with a clinically diagnosed condition, such as Schizophrenia, then the risk posed by the app may be higher than if the person has mild anxiety. Similarly, if the app targets children and adolescents, then the potential risk associated with the app may be higher than if it targeted adults with no impairment or mental health diagnosis.

*For the purposes of this report, either minors (younger than 18 years of age) or adults with a moderate or severe level of functional impairment that causes them to need support from a caregiver are considered to be part of a* ***vulnerable population****.*

*The information requested in these questions can be found within the app/app website.*

**5. Is the app intended for use by adults who may have an illness or disability that impacts their decisionmaking ability? Severe autism or severe dementia are examples of illnesses or disabilities that may affect decisionmaking ability.**

*For the purposes of this assessment, the population that may have an illness or disability that impacts their decision making ability is included in the "vulnerable population" group. Adults can usually make medical decisions, such as consenting to treatment, on their own. In some cases, adults need someone else to make medical decisions for them if their decisionmaking ability is affected by an illness or disability. This is sometimes referred to as “substituted consent.” A higher level of evidence/protection is needed for the use of specific apps under these circumstances.*

- - Yes
  - No
  - Unable to assess

**6. Does the app claim that it is intended for use by minors (i.e., people younger than 18 years of age)?**

*For the purposes of this assessment, this population is included in the "vulnerable population" group. The app is “intended for use” by a population if it is clearly specified on the app or website that this population is its target user group. Do not look at the App Store Age Ratings or Terms and Conditions for this information.*

- - Yes, for use by minors
  - Yes, for use by both minors and adults
  - No, not for use by minors
  - Unable to assess

**7. Does the app claim to provide standalone treatment for any mental health condition?**

*A standalone treatment might be some form of psychotherapy or other any other medical care provided by the app independently, rather than in conjunction with a licensed healthcare practitioner.*

*Functionality, such as mindfulness training, skills training, symptom or mood tracking, and social support, would not be considered standalone treatment.*

- - Yes, the app provides standalone treatment
  - No, the app does not provide standalone treatment
  - Unable to assess

**C. Evidence**

It is important that the app has a solid clinical foundation. If the app targets a vulnerable population, the burden of evidence to prove efficacy and safety is greater.

*The information requested in these questions can be found on the app website (look at customer testimonials or resources/blog for anecdotal information) and on PubMed (search PubMed as outlined in the training document). Make sure to save all the references in the app review document.*

**8. Has the app been evaluated for efficacy/effectiveness through a scientifically validated study?**

*This can be determined by evaluating whether a research study about the app has been published in a peer-reviewed journal. The research should be specific to the mental health component of the app.*

- - Yes, there is strong research support for the app (i.e., at least one published paper in a peer-reviewed journal that uses a randomized trial that shows efficacy or effectiveness)
  - Yes, there is some research support for the app (i.e., at least one published paper in a peer-reviewed journal that uses single-case design, quasi-experimental methods demonstrating efficacy)
  - No, there is no research support for the app

**9. Is the app based on or does it use an evidence-based strategy, such as Cognitive Behavioral Therapy (CBT), Dialectical Behavior Therapy (DBT), or evidence-based guidelines?**

*Evidence-based medicine aims to assess the strength of proof behind medical interventions in terms of risks and benefits and, therefore, can be used to inform clinical decisionmaking on both an individual and a population basis.*

- - Yes, the app reports using an evidence-based strategy/guidelines to achieve its goals
  - No, the app does not report the use of evidence-based strategy/guidelines to achieve its goals (or there are no goals described)
  - Unable to assess

**D. Linkage to Care**

This category evaluates the linkages to healthcare providers, caregivers. This information can be found within the app/app website.

**10. Does the app facilitate remote monitoring of the patient or send alerts to a clinician/clinical care team or caregiver?**

*This question seeks to assess whether a healthcare provider or caregiver can monitor the user’s health data in real time AND/OR receive alerts triggered by certain pre-specified high-risk events. Functionality to export data into a file or an email does not count as facilitation of remote monitoring.*

- - Yes
  - No
  - Unable to assess

**10.a. If YES, please specify *who* can monitor the health of the patient, either through alerts or by other means?**

- - Healthcare provider only
  - Caregiver only
  - Both healthcare provider and caregiver
  - Other
  - Not applicable

**E. Access to Crisis Services**

This section evaluates whether the app provides access to emergency sources of information.

*This information can be found within app/app website. There should be a caregiver or healthcare provider interface within the app. Exporting data into a file or an email would not be considered facilitation of remote monitoring.*

**11. Does the app provide users with information about resources that can be reached in case of an emergency? This information should be available on the mobile app. Reviewers should not look for this information on the website.**

- - Yes (e.g., a crisis hotline, 911, nearest emergency room services.)
  - No, the app provides no information on emergency services
  - Unable to assess

**12. Evidence and Linkage to Care: If the app can be used by individuals that require substituted consent OR by minors, then is consent sought from either a caregiver/parent/ legal guardian?**

*This should be assessed at the time of creating a login for the app.*

- - Yes
  - No
  - Unable to assess
  - Not applicable

**Risk Assessment**

| **What is the Risk Category for the App?**  *A lower number signifies a lower risk level.*  **Risk Level 1:** Apps rated as Risk Level 1 do not target a vulnerable population AND do not provide standalone treatment. These are likely to be apps that provide functions such as mindfulness, meditation, and wellness resources. No specific safety checks are needed for these apps.   - - **Risk Level is 3**: Response to EITHER Question 5 or Question 6 is **Yes** AND response to Question 7 is **Yes**, *go to Safety Assessment Step 3*   - **Risk Level is 2**: Response to EITHER Question 5 or Question 6 is **Yes** OR response to Question 7 is **Yes**, *go to Safety Assessment Step 3*   - **Risk Level is 1**: None of the above, *go to Safety Assessment Step 3* |
| --- |

**Safety Assessment**

**Step 1: Preliminary Safety Assessment for Risk Levels 2 & 3**

| **Risk Level 2** | - - [If responses to Questions 9-11 are **Yes** AND response to Question 10 is **Yes** or **No**, supporting evidence-base available] | **PRELIM SECURITY CHECK PASSED,** *go to Safety Assessment Step 2* |
| --- | --- | --- |
|  | **Else** | **PRELIM SECURITY CHECK FAILED,** *go to Safety Assessment Step 2* |
| **Risk Level 3** | - - [If responses to Questions 8-11 are **Yes**, supporting evidence-base available] | **PRELIM SECURITY CHECK PASSED,** *go to Safety Assessment Step 2* |
|  | **Else** | **PRELIM SECURITY CHECK FAILED,** *go to Safety Assessment Step 2* |

**Step 2: Safety Assessment for Vulnerable Population**

| If response to Question 5 and/or Question 6 is Yes or Unable to Assess,   - - If response to Question 12 is **Yes**, **then SAFE for vulnerable populations,** *go to Safety Assessment Step 3*   - If response to Question 12 is **No or Unable to Assess** **then NOT SAFE for vulnerable populations,** *go to Safety Assessment Step 3*   **else**  *go to Step 3 under Safety Assessment* |
| --- |

**STEP 3: Safety Assessment**

| **RISK LEVEL** | **PRELIM SAFETY CHECK** | **VULNERABLE POPULATION** | **APP SAFETY** |
| --- | --- | --- | --- |
| **Risk Level 1** | N/A | N/A or SAFE for Vulnerable Populations | Passed Safety Check |
|  | N/A | Not Safe for Vulnerable Populations | Failed Safety Check |
| **Risk Level 2** | PASS | N/A or SAFE for Vulnerable Populations | Passed Safety Check |
|  |  | Not Safe for Vulnerable Populations | Failed Safety Check |
|  | FAIL | -- | Failed Safety Check |
| **Risk Level 3** | PASS | N/A or SAFE for Vulnerable Populations | Passed Safety Check |
|  |  | Not Safe for Vulnerable Populations | Failed Safety Check |
|  | FAIL | -- | Failed Safety Check |

**Section 2: Function**

This section is focused on descriptive aspects, such as accessibility, usability, privacy/security, AI, cultural competence, and informed consent.

**A. Accessibility Features**

Accessibility features are meant to make the app easier to navigate, especially for those with disabilities. Common accessibility features include text-to-speech, closed-captioning, and keyboard shortcuts.

**1. Which of the phone’s accessibility features work within the app?**

*Reviewers will have to activate accessibility features on their phone under Settings in order to test this.*

**Select all that apply:**

▢ Text adjustment feature

▢ Colorblind color scheme feature

▢ Text-to-speech feature

▢ None of the phone’s accessibility features work in the app

▢ Unable to assess, please provide a reason:____________________________________

**2. Are there additional accessibility features that are provided by the app?**

*These are accessibility features that are provided within the app but cannot be set as an option under Settings.*

**Select all that apply:**

▢ Adaptation of audio/video content with transcriptions or captions

▢ Tapping and other gestures are configurable

▢ Contrast text coloring in the content

▢ Screen reader

▢ No additional accessibility features provided by the app

▢ Other features not provided here (please specify): ___________________________________

▢ Unable to assess, please provide a reason:________________________________________

**B. App Information**

General info about the app and user ratings.

*The information requested in these questions can be obtained by going to the appropriate app store using your browser.*

**3. Does the app work on Apple(iOS)?**

- - Yes
  - No

**4. What are the number of reviews on the iOS App Store?**

*The iOS App Store can be accessed through a browser on your phone, tablet, or computer.*

________________________________________________________________

**5. What is the app rating (number of stars) on Apple Store?**

*This provides users’ perspectives of the app.*

________________________________________________________________

**6. Does the app work on Android?**

- - Yes
  - No

**7. What are the number of reviews on Google Play Store.**

________________________________________________________________

**8. What is the app rating (number of stars) on Google Play Store?**

*The Google Play Store can be accessed through a browser on your phone, tablet, or computer.*

______________________________________________________________

**C. Costs**

App costs could be upfront, in the form of a monthly/annual subscription, or through freemium services that require in-app purchasing. Some apps require payment from the user, while others are reimbursed by the healthcare provider or insurance.

*The information requested in these questions may be found in the app, in the App Store, and on the app site.*

**9. What is the business model for the app? More than one option may be applicable here. An app that provides a free trial for a limited duration is not considered to be free.**

*If the app provides a payment gateway to pay a therapist or healthcare provider, those costs should not be included.*

▢ Free (no upfront fee for the app; additional packages/services may be offered for a fee)

▢ Upfront fee (a onetime cost for accessing the app; additional packages/services may be offered for additional fees)

▢ In-app purchases (additional packages/services available for a fee in addition to or in lieu of an upfront cost, e.g., concierge services)

▢ Subscription (payment for services on a monthly/quarterly/annual basis)

▢ Reimbursed by healthcare providers/insurers/employers

▢ Other, please specify: ________________________________________________

**10. Does the app provide a free or freemium model? An app that provides a free trial for a limited duration is not considered to be free.**

- - Free
  - Freemium
  - No free or freemium version

**11. What is the estimated annual cost of the app for the paid version?**

*If the app provides a free or freemium model, then select “Not applicable” in the list below.*

- - Under $50
  - $50-250
  - Over $250
  - Not applicable

**12. If the app includes paid service(s), does it provide CPT code(s) for insurance reimbursement?**

*Current Procedural Terminology (CPT) are unique 5-digit codes for medical services or procedures assigned by the American Medical Association which are used throughout the U.S. medical system. Insurance companies decide on which CPT codes can be used for insurance reimbursement.*

- - Yes
  - No or the app has no paid services
  - Unable to Assess, please provide a reason:__________________________

**D. Organizational Credibility**

Organizational credibility is meant to determine whether the app comes from a trusted source.

**13. Who is the developer of the app?**

*This information can be found on the app website or in the Terms of Service on the app. More than one response may apply. For example, two or more institutions may be involved in the development of the app; or, for example, if an app was developed by Johns Hopkins University, then “Non-profit institution” and “Academic institution” would apply.*

▢ Government

▢ For-profit company

▢ Non-profit institution

▢ Healthcare institution

▢ Academic institution

▢ Insurance company

▢ Independent developer/s

▢ Unable to assess

▢ Other, please specify: ________________________________________________

**14. Does the app have any consumer bureau complaints or lawsuits pending?**

*This information can be found here: Better Business Bureau: https://www.bbb.org/us/ca/san-francisco/profile/mobile-apps/calmcom-inc-1116-877519/complaints ; Google Search: <Consumer Complaints> <app name> <app/mobile app>; Google Search: <law suits> <app> <app/mobile app>*

- - Yes
  - No
  - Unable to assess

**E. Evidence & Clinical Foundation**

The information requested in these questions can be found on the app website.

**15. Does the app appear to do what it claims to do?**

*Look at the website to see the claims made by the app and compare them to what you see in the app.*

- - Yes, the app provides the functionality it claims on its website
  - The app provides some of the functionality it claims on its website
  - No, the app does not provide the functionality it claims on its website
  - Unable to assess

**F. Privacy & Security**

Security is about the safeguarding of data and unauthorized access of the app. Privacy is about the safeguarding of the user’s identity.

*The information requested in these questions should be available in the app in the Terms & Conditions or Privacy & Security agreement. Do not look at information provided on the website.*

**16. Does the app claim it meets HIPAA [or analogous national standard for protected health information (PHI)]?**

- - Yes
  - No

**17. Does the app claim it meets COPPA [or analogous national standard for protected health information (PHI) for minors younger than 13 years of age]?**

*The Children's Online Privacy Protection Act (COPPA) applies to the online collection of personal information by persons or entities under U.S. jurisdiction about children younger than 13 years of age.*

- - Yes
  - No

**18. Does the app report sharing or selling of data for research or commercial purposes?**

*Information used internally within the app itself to make better recommendations to the user does not count as sharing or selling. Examples of apps that may be sharing or selling data include those that provide evidence based drug and dosage information, clinical decision support systems where you share conditions, symptoms and medication information, medication adherence apps, etc.*

- - Yes
  - No
  - Unable to assess

**19. If your response to question 18 is “YES”, please answer the following question:**

**If the app reports sharing or selling for research or commercial purposes, are the data de-identified?**

*This question is targeted at understanding if the data is de-identified or not. Information used internally, within the app itself, to make better recommendations to the user does not count as sharing or selling.*

- - Yes, the app reports that data is de-identified
  - No, the app does not report data is de-identified
  - Unable to assess

**20. If the app has the capability to read/write to an electronic health record management system (EHRs) or other healthcare systems, does it use industry standards for secure interoperability (e.g., FHIR, SMART, OAuth 2.0, TLS 1.2)?**

*This information may need to be deduced. If the app has existing integrations with known EHRs such as EPIC, it can be concluded that it follows industry standards. This information may also need to be identified through a review of the app developer webpage.*

- - Yes, the app uses industry standards for interoperability
  - No, the app does not use industry standards for interoperability
  - Not applicable, because it does not read/write to EHRs
  - Unable to assess

**G. Informed Consent**

Informed consent is permission granted by the user after information about the potential use or disclosure of the information collected by the app is provided. Most apps tend to have a disclosure list that is long and hard to understand. There are best practices for ensuring that users understand exactly what they are agreeing to before they “Agree” to the app’s privacy and security practices. The following questions evaluate whether the app follows these best practices.

*The information requested in these questions can be found within the app.*

**21. Please assess the level of informed consent enabled by the app.**

*Providing consent is often one of the first things users are asked to do after downloading an app. Traditional written consent forms (e.g., Terms of Service, Privacy and Security agreement) are often too long, difficult to understand, and overly focused on legal concerns. It is important that apps enable informed consent for prospective users in a manner that is easy to understand.*

- - No informed consent
  - Does not simplify informed consent
  - Average: Focuses on the essential by a) providing a narrative focused on the most salient information, b) limiting concepts to one per screen, and c) following national plain language and health literacy guidance
  - Good: Focuses on the essential and organizes content deliberately by a) prioritizing key words/concepts presented on each screen, b) providing information tiers for conceptual elaboration, and c) enabling participants to navigate to their desired level of detail
  - Excellent: Focuses on the essential, organizes content deliberately, and encourages engagement through interactive elements

**22. What is the format of the data privacy and security consent process followed in the app?**

*An opt-in consent process requires* ***explicit consent from the user*** *before the collection and processing of their personal data. It refers to a* ***positive action*** *taken by the user indicating that they agree to the use of their personal data. An opt-out consent process* ***does not require the user’s consent*** *prior to the collection and processing of their personal data. It refers to the process by which users* ***withdraw their consent*** *to the use of their personal data.*

- - Opt-in for data to be shared, the default choice is opt-out
  - Opt-out of data sharing, the default choice is opt-in
  - No choice to opt-out of data sharing, without explicit consent you can’t use the app
  - Unable to assess

**H. Cultural Competence**

Cultural competence is defined as the ability to understand, appreciate, and account for different cultures or belief systems based on race, ethnicity, income strata, religious beliefs, etc. It is important to assess if the app captures these differences and whether it provides personalized care that takes these cultural differences into account.

*The information requested in these questions can be found within the app or on the app website.*

**23. Does the app report developing and testing the app for specific cultural group/s?**

*According to the U.S. Department of Health and Human Services (HHS), "cultural competence is the integration and transformation of knowledge about individuals and groups of people into specific standards, policies, practices, and attitudes used in appropriate cultural settings to increase the quality of services; thereby producing better outcomes.” For this assessment, groups with lived experiences such as pregnant teens and survivors of gender-based violence would be considered to be a specific cultural group.*

- - Yes
  - No
  - Unable to assess
  - Other, please specify: ________________________________________________

**24. If the app reports developing and testing the app for specific cultural groups, is there published documentation (e.g., website, papers) of the process taken to incorporate information that is specific to specified cultures?**

**Select all that apply:**

- - Website
  - Documentation (on the app or website)
  - Published scientific papers
  - Unable to assess
  - Other, please specify: ________________________________________________
  - Not applicable

**If the app integrates culturally specific groups, please name the groups.**

________________________________________________________________

**25. Is gender-inclusive language employed? When asking the user’s gender, is there an option to self-describe, an option to decline to answer, use of scientifically correct terms for gender (e.g., man, woman, nonbinary)?**

*This information can be found while assessing the app and is generally encountered when a user creates an account on the app.*

**Select all that apply:**

▢ An option for use of personal pronouns (e.g., they/them, he/his, she/her)

▢ An option to specify another gender

▢ An option to decline to answer

▢ Use of scientifically correct terms for gender, such as man, woman, non-binary

▢ Unable to assess

▢ Not applicable

**26. If the app was tested in a study, what was the percentage of non-white participants?**

*For clinical trials, this information may be found on the clinicaltrials.gov page of the trial under Study Results (include “Other” as non-white). If the study was a usability or other type of study, this information may be found on the developer website.*

- - Less than 30%
  - Between 30 – 50%
  - More than 50%
  - No disaggregate data available
  - No information available

**I. Usability**

Usability can be described as the capacity of a system to provide a condition for its users to perform the tasks safely, effectively, and efficiently. It is important that the experience be engaging and pleasing, otherwise users are likely to stop using the app.

*The information requested in these questions can be found within the app.*

**27. Does the app work offline?**

*Choose* **Yes** *if parts of the app or the entire app work offline.*

- - Yes
  - No
  - Unable to assess

**28. Performance: How accurately/fast do the app features (functions) and components (buttons/menus) work?**

- - App is broken; no/insufficient/inaccurate response (e.g., crashes/bugs/broken features)
  - Some functions work, but app lags or contains major technical problems
  - App works overall; some technical problems need fixing or app is slow at times
  - Mostly functional with minor/negligible problems
  - Perfect/timely response; no technical bugs found or contains a ‘loading time left’ indicator, if relevant

**29. What languages are supported by the application?**

*This information can be found in the App Store.*

**Select all that apply:**

▢ English

▢ Spanish

▢ French

▢ German

▢ Other, please specify: ________________________________________________

**30. Customization: Does the app allow customization of settings and preferences by the user (e.g., sound, content, notifications)?**

- - Does not allow any customization or requires setting to be input every time
  - Allows little customization and that limits the app’s functions
  - Basic customization to function adequately
  - Allows numerous options for customization
  - Allows complete tailoring to the user’s characteristics/preferences, remembers all settings

**31. Target group: Is the app content (visuals, language, design) appropriate for the target audience?**

- - Completely inappropriate, unclear, or confusing
  - Mostly inappropriate, unclear, or confusing
  - Satisfactory, but not specifically designed for the target audience, may be inappropriate/unclear/confusing at times
  - Designed for the target audience, with minor issues
  - Designed specifically for the target audience; no issues found

**32. Layout:** **Is the arrangement and size of buttons, icons, menus, and content on the screen appropriate?**

- - Very bad design: cluttered, some options impossible to select/locate/see/read
  - Bad design: random, unclear, some options difficult to select/locate/see/read
  - Satisfactory: few problems with selecting/locating/seeing/reading items
  - Mostly clear: able to select/locate/see/read items
  - Professional: simple, clear, orderly, logically organized

**33. Graphics: How high is the quality/resolution of graphics used for buttons, icons, menus, and content?**

- - Graphics appear amateur, very poor visual design: disproportionate, stylistically inconsistent
  - Low quality/low resolution graphics, low quality visual design: disproportionate
  - Satisfactory quality graphics and visual design: generally consistent in style
  - High quality/resolution graphics and visual design: mostly proportionate, consistent in style
  - Very high quality/resolution graphics and visual design: proportionate, consistent in style throughout

**34. Visual appeal: How good does the app look?**

- - Ugly/unpleasant to look at: poorly designed, clashing, mismatched colors
  - Bad: poorly designed, bad use of color, visually boring
  - Satisfactory: average, neither pleasant nor unpleasant
  - Pleasant: seamless graphics, consistent and professionally designed
  - Beautiful: very attractive, memorable, stands out; use of color enhances app features/menus

**35. Does the app have advertising?**

- - Yes
  - No

**36. If app has advertising, is the advertising intrusive and distracting?**

- Advertising is neither intrusive nor distracting
- The advertising is both intrusive and distracting
  - Not applicable

**37. Ease of use: How easy is it to learn how to use the app; how clear are the menu labels, icons, and instructions?**

- No/limited instructions, menu labels and icons are confusing, complicated
- Takes a lot of time or effort
- Takes some time or effort
- Easy to learn (or has clear instructions)
- Able to use app immediately, intuitive and simple (no instructions needed)

**38. Navigation: Does moving between screens make sense? Does the app have all necessary links between screens?**

- No logical connection between screens at all/navigation is difficult
- Understandable after a lot of time/effort
- Understandable after some time/effort
- Easy to understand/navigate
- Perfectly logical, easy, clear and intuitive screen flow throughout, and/or has shortcuts

**39. Gestural design: Do taps/swipes/pinches/scrolls make sense? Are they consistent across all components/screens?**

- Completely inconsistent/confusing
- Often inconsistent/confusing
- Satisfactory with some inconsistencies/confusing elements
- Mostly consistent/intuitive with negligible problems
- Perfectly consistent and intuitive

**40. Content: Is the app copy well written and relevant to the goal and/or topic of the app?**

- - There is no information within the app
  - Irrelevant/inappropriate/incoherent/incorrect
  - Barely relevant/appropriate/coherent, may be incorrect
  - Satisfactory with respect to relevance/appropriateness/coherence and appears to be correct
  - Relevant/appropriate/coherent/correct
  - Highly relevant/appropriate/coherent/correct
  - Not applicable

**41. Is there any evidence to show the average duration of use of the app by users?**

*This information may be available on the app website or in a paper published in a scientific journal. Do not look at user reviews on the app website, in the App Store, or in news articles.*

- - On average, users have engaged with the app for 30 days or less
  - On average, users have engaged with the app for more than a month but less than 6 months
  - On average, users have engaged with the app for more than 180 days
  - No evidence available

**J. Remote Monitoring**

Remote patient monitoring enables monitoring of patients outside of conventional clinical settings through real-time access to patient health data. The patient’s provider may have a schedule to monitor patient data or may receive alerts about their patient's health.

*The information requested in these questions can be found within the app/app website.*

**42. If remote monitoring is feasible, how does the provider access the data?**

- - Provider has access to patient information through the app
  - Provider has access to the patient information through the EMR
  - Provider has no access to patient information
  - Not applicable
  - Unable to assess
  - Other, please specify: ________________________________________________

**43. Does the app provide alerts to the provider to notify them of a clinical event that may require action on their part?**

*Two-way messaging capability with a healthcare provider or concierge may be considered to be an alerting mechanism.*

- - Yes
  - No
  - Unable to assess

**44 . Can the app share data with wearables like Apple Watch and Fitbit?**

*This option will either pop up on the app during sign-up or be mentioned on the app developer’s website.*

- - Yes
  - No
  - Unable to assess

**K. Access to Crisis Response Services**

This section evaluates whether the app provides access to emergency sources of information.

*The information requested in this question can be found within the app or on the app website.*

**45. Does the app connect the user automatically to resources in case of a crisis situation or emergency?**

- - Yes
  - No
  - Unable to assess

**L. Artificial Intelligence (AI)**

Apps often use artificial intelligence (AI) for a variety of tasks including risk-prediction, differential diagnoses, and personalization of health content. The following questions are important because we want to gauge the potential of the app to cause harm and also to determine whether the app’s algorithms are being updated based on user input.

*The information requested in these questions can be found in the app, on the app website, or from evidence found through a literature search.*

**46. Does the app use AI? If yes, please respond to questions 47 and 48.**

- - Yes
  - No

**47. How is AI being used in the app?**

*For example, AI may be used in the app to automate simple tasks, such as scheduling, or it may be used for more complex functionality, such as to personalize chatbots.*

________________________________________________________________

**48. Is there any evidence to suggest that the app uses data from user interactions to improve precision on AI models?**

*For example, after an interaction, the app may ask the user for feedback on the automated response from the app to see if it was appropriate. If the response was not correct, the app may ask for further feedback from the user so the algorithms can be refined.*

- - Yes, there is evidence to suggest that the AI models are updated based on feedback
  - No, there is no evidence to suggest that the AI models are updated based on feedback
  - Unable to assess

**Section 3: Mental Health App Features**

This section attempts to capture the features specific to mental health present in the app. This is not meant to be an exhaustive list. Only some of these features may apply to the app being reviewed.

*The information requested in these questions can be found in the app, on the app website or in published literature.*

**1. List mental health symptom(s) and/or condition(s) addressed by the app.**

________________________________________________________________

**2. Please answer the following questions about the app features.**

|  | Yes | No | Unable to assess |
| --- | --- | --- | --- |
| a. Does the app facilitate text messaging interactions with healthcare therapists, coaches, or other providers via the app? |  |  |  |
| b. Does the app facilitate audio chat interactions with healthcare therapists, coaches, or other providers via the app? |  |  |  |
| c. Does the app facilitate video chat interactions with healthcare therapists, coaches, or other providers via the app? |  |  |  |
| d. Does the app facilitate teletherapy services via the app? |  |  |  |
| e. Does the app facilitate group therapy services via the app? |  |  |  |
| f. Does the app provide live support to a coach or counselor via the app? |  |  |  |
| g. Does the app provide concierge mental health services via the app? Concierge services are personalized services patients can choose based on what they think works best for them. Some apps may provide acute concierge services to include additional help during times of crisis or high stress. |  |  |  |

**3. Does the app provide a direct connection to 988 or other hotlines?**

- - Yes, and the connection works
  - Yes, but the connection does not work
  - No
  - Unable to assess

**4. Are the following functionalities supported by the app?**

The following questions ask you to rate the functionality as either “comprehensive” or “not comprehensive.”

**Comprehensive:** Broad range of functionality that meets or exceeds the needs of the user and allows for flexibility

**Not comprehensive:** Rudimentary functionality that may not meet the needs of the user or provides limited choices and lacks flexibility

|  | **Comprehensive** | **Not Comprehensive** | **Not applicable** |
| --- | --- | --- | --- |
| **Mindfulness:** Mindfulness is a therapeutic technique that includes elements of relaxation, breathing, and body exercise. It also includes techniques, such as meditation, guided positive imagery, grounding exercises, or progressive muscle relaxation. |  |  |  |
| **Journaling:** Journaling can help users manage anxiety, reduce stress, and cope with mental health challenges. It can provide users with a way of identifying negative thoughts and behaviors and highlight positive aspects in their lives. |  |  |  |
| **Psychoeducation:** Psychoeducation may be defined as the education of a person with a psychiatric disorder regarding the symptoms, treatments, and prognosis of that illness. Psychoeducation may also be targeted to the caregiver, family member, or loved one of the patient. Psychoeducation should be a brief personal intervention by a healthcare provider upon first diagnosis; however, sometimes, only written materials or online resources are provided to the patient. In the worst-case scenario, patients receive no Psychoeducation from their provider. |  |  |  |
| **Skill building:** Skill building includes techniques for recognition of signs and symptoms of a problem, self-advocacy, stress management, emotional regulation, relapse prevention, promotion of sleep hygiene, etc. The app may include tips and advice on dealing with negative emotions and with behavior change. Skill building may require repetition, positive reinforcement, modeling, and practice. |  |  |  |
| **Screening:** Historically, mental health care has relied on structured patient interviews and self-reported questionnaires for diagnosis. These can be used for self-evaluation, reporting, or to decide whether a patient should engage with a mental health professional. Please indicate if industry-validated screening questionnaires (e.g., GATT, PHQ-4, PHQ-9) are provided by the app. The standardized screening questionnaires used will generally be mentioned on the app developer’s website. |  |  |  |
| **Safety planning:** Safety planning is designed to help individuals respond to escalation of suicidal thoughts and feelings, giving them a tailored list of coping behaviors and social support to use until the risk passes. |  |  |  |
| **Sleep Hygiene:** This includes features for promoting sleep hygiene, such as sleep diaries, lifestyle tracking, alarms, data synchronization with a wearable device (e.g., FitBit, Apple Watch), etc. |  |  |  |
| **Automated Chatbots:** Chatbots include conversational agents that can provide virtual therapy and/or social support. |  |  |  |
| **Family/Caregiver Support:** Caregivers take care of the day-to-day needs of patients. Apps can help caregivers monitor the mental state of the patient, identify the early signs of illness, track relapse and deterioration, and help the patient access services. The family/caregiver may also supervise treatment and provide emotional support to the patient. |  |  |  |
| **Social and peer group interaction:** Individuals with mental health conditions may find it hard to engage socially and may have a small social network/peer group. Apps may provide peer group interactions and/or community support discussion boards for such individuals. |  |  |  |
| **Gamification:** Gamification is the application of typical elements of game playing (e.g., point scoring, competition with others, rules of play) to other areas of activity. Gamification is used to encourage and reward positive changes. |  |  |  |
| **Personalization:** Personalized treatment entails the selection of a therapy or treatment protocol based on a patient’s profile, which may increase the likelihood of a successful outcome. Personalization can be used to tailor interactions initially, when the user starts to use the app, and also on a more regular basis. It is usually based on user information collected by the app, such as what activities produce positive emotions (e.g., physical exercise, talking to a friend). |  |  |  |
| **Self-Monitoring:** Self-monitoring apps allow users to engage in symptom monitoring, mood tracking, and progress tracking through a treatment program, such as one for substance abuse, depression and anxiety, etc. |  |  |  |
| **Medication Adherence:** Does the app support patients in their use of medication(s)? This could be through alerts, notifications, or other means. |  |  |  |
| **Medication Delivery:** Does the app support physical delivery of medications to patient(s)? |  |  |  |

**5. Please add any features supported by the app that are not listed above.**

**________________________________________________________________**

**Post Administrative Questionnaire**

This section contains some details about the app and evidence being reviewed.

**1. Include all links to references for evidence (citations on PubMed, systematic reviews, websites, etc.) here.**

**2. Based on your review of the app, do you think it would have been useful to have some training or a tutorial about how to use it?**

*This is your subjective assessment based on your usage of the app.*

- - Yes, there was training available, and I needed it to use the app
  - Yes, there was training available, but I didn't need it to use the app
  - No, there was no training available, but I needed it to use the app
  - No, there was no training available, and I didn’t need it to use the app
  - Don’t know

**3. Does the app have any help-related documentation available in the app itself (e.g., tooltips, general help)?**

*Base your answer on what was available on the app itself.*

*In-app help usually comes in three forms: 1) a separate page or pages of help within the app, with instructions; 2) popups that provide contextual help, displaying instructions relevant to the specific task that the user is attempting; 3) descriptions of app features of interest to the user. If at least two of these features is present, the help-related documentation would be considered to be comprehensive.*

- - Yes, there was comprehensive help available on the app
  - Yes, there was some help available on the app
  - The app help button took me to the website
  - No help was available on the app

**4. Does the app have a dedicated website that provides information about the app?**

*This may be part of a company website or a standalone website focused solely on the app.*

- - Yes
  - No
  - Unable to assess

**5. Does the app have any help-related documentation available on its website?**

- - Yes, there was comprehensive help available
  - Yes, there was some help available
  - No, help was not available on the app

**6. Please provide a subjective evaluation of the app in your own words.**

- Do you think the assessment conducted here matches your subjective assessment? If not, please provide additional details.
- Do you think the risk posed by the app is captured accurately here?
- Do you think the app is technically sound?
- Do you think the therapeutic content provided by the app is rudimentary or substantive?
- Can this app help those that may be experiencing mental or behavioral health challenges?

________________________________________________________________

**7. Do you think the app could cause harm or have a negative impact on the user? Please elaborate.**

________________________________________________________________

**8. Do you think the subjective risk assessment matches the risk assessment calculated in Section 1? Please elaborate.** ________________________________________________________________

**9. Any additional comments?**

________________________________________________________________

________________________________________________________________

**10. Please note the time taken to complete your assessment using this framework.**

________________________________________________________________

**Supplementary Table D: List of Apps reviewed**

| **Round** | **Number of apps reviewed** | **Apps Reviewed** |
| --- | --- | --- |
| Pre-Pilot | 10 | Breathe, Think, Do; BUP; Daylio; Replica; Sleepio; SuperBetter; T2 Mood Tracker; The Safe Place; Woebot; Youper |
| Pilot Round 1 | 5 | Narcolepsy Monitor; WeAreMore; Awell; Pursue Care; MDLIVE |
| Pilot Round 2 | 5 | StressScan; Mental Health Tests – Mind Diagnostics; Change Scope; Diet or Disorder; FearTools |
| Pilot Round 3 | 4 | Self-Harm Recovery; Schizophrenia Health Storylines; Staney-Brown Safety Plan; PsychSurveys |
| Pilot Round 4 | 3 | Calm; Joyster; Simple Habit (Sleep, Meditation) |
| Pilot Round 5 | 8 | Tourette Syndrome; Pattern: Correllate, Health Diary, Mood Tracker; Mental Health Test; Cognitive Stimulation Questions; Quitzilla; Njoy; Narcolepsy Disorder; ShutEye: Sleep Tracker |
| Pilot Round 6 | 10 | Bipolar Disorder; Mental Health Tracker: Disorders Test; Quirk CBT; Reflectly – Journal and AI Diary; Meditopia; Relaxing Music 2021; Nina App; Breeze: Mood Tracker, Diary; Waking Up: Guided Meditation; PepTalk Motivation |

**Glossary**

| **Term** | **Explanation** |
| --- | --- |
| Artificial Intelligence (AI) | The ability of a computer to do tasks that are usually done by humans. |
| Caregiver | A family member or helper who regularly looks after a child or a sick, elderly, or disabled person. |
| Cultural Competence | The ability to understand, appreciate, and account for different cultures or belief systems based on race, ethnicity, income strata, religious beliefs, etc. |
| Functional Impairment | Limitations owing to illness(es) that cause an inability to carry out certain functions in one’s daily life. |
| Gamification | Application of typical elements of game playing (e.g., point scoring, competition with others, badges and awards) to other areas of activity to encourage engagement with a product or service. |
| Informed Consent | Permission granted in the knowledge of the possible consequences, typically that which is given by a patient to a doctor for treatment with full knowledge of the possible risks and benefits. |
| Vulnerable Population | For the purposes of this report, minors, developmentally disabled adults, and older adults who have become incapacitated and require a legal guardian. |
